# Mistaken identity? Visual similarities of marine debris to natural prey items of sea turtles

**DOI:** 10.1186/1472-6785-14-14

**Published:** 2014-05-09

**Authors:** Qamar A Schuyler, Chris Wilcox, Kathy Townsend, B Denise Hardesty, N Justin Marshall

**Affiliations:** 1 School of Biological Sciences, University of Queensland, St. Lucia, Australia; 2 Wealth from Oceans Flagship Marine and Atmospheric Research, Commonwealth Scientific and Industrial Research Organization, Hobart, Australia; 3 Moreton Bay Research Station, University of Queensland, Dunwich, Australia; 4 Queensland Brain Institute, University of Queensland, St. Lucia, Australia

**Keywords:** *Chelonia mydas*, Chromatic space, *Eretmochelys imbricata*, Marine debris, Vorobyev-Osorio model

## Abstract

**Background:**

There are two predominant hypotheses as to why animals ingest plastic: 1) they are opportunistic feeders, eating plastic when they encounter it, and 2) they eat plastic because it resembles prey items. To assess which hypothesis is most likely, we created a model sea turtle visual system and used it to analyse debris samples from beach surveys and from necropsied turtles. We investigated colour, contrast, and luminance of the debris items as they would appear to the turtle. We also incorporated measures of texture and translucency to determine which of the two hypotheses is more plausible as a driver of selectivity in green sea turtles.

**Results:**

Turtles preferred more flexible and translucent items to what was available in the environment, lending support to the hypothesis that they prefer debris that resembles prey, particularly jellyfish. They also ate fewer blue items, suggesting that such items may be less conspicuous against the background of open water where they forage.

**Conclusions:**

Using visual modelling we determined the characteristics that drive ingestion of marine debris by sea turtles, from the point of view of the turtles themselves. This technique can be utilized to determine debris preferences of other visual predators, and help to more effectively focus management or remediation actions.

## Background

Sea turtles, like many other marine taxa, are increasingly prone to marine debris ingestion and associated problems [1]. Despite many studies recording instances of debris ingestion e.g. [2,3], little is known about the cues that attract turtles to eat plastic debris. The predominant hypotheses are that 1) turtles, as opportunistic feeders, simply consume items in proportion to what they encounter in the environment, including plastics; and 2) that turtles feed on plastic because of its similarity to prey; particularly jellyfish [4,5]. Though the proportion of gelatinous prey in a turtle’s diet varies depending on the life stage and the species of the turtle, all species do target these prey at some stage of their lives [6,7].

Turtles are primarily visual predators. Research indicates that loggerhead turtles have limited ability to find food based on chemical stimuli alone [8], which may explain why they are primarily caught during the day on longline fishing lines, and rarely at night [9]. Similarly, when presented with both chemical and visual cues, leatherback turtles responded exclusively to visual cues [10]. Therefore, the visual similarity between plastic bags and jellyfish can cause confusion even in the absence of chemical stimuli associated with food sources. Loggerhead sea turtles have been shown to approach plastic bags in a similar manner to gelatinous prey, indicating that they use visual characteristics to select their food [11].

The spectral sensitivity of an animal depends not only on its photopigments, but also on the transmissivity of the ocular media and, in the case of turtles, of the oil droplets associated with the cones. Turtles have a well-developed visual system with at least three different photopigments, indicating the ability to see colour [12]. The visual system of sea turtles is similar to that of fresh water turtles; however, the sea turtles’ visual pigments are slightly shifted towards the shorter wavelengths, due to the differences in spectral characteristics of the waters in which the different animals live [13]. Sea turtles generally inhabit clearer, oceanic waters, whereas fresh water contains many dissolved organics and sediments, shifting the maximum light transmission to longer wavelengths [13-15]. The bulk of sea turtle vision studies to date have been conducted on green (*Chelonia mydas*) and loggerhead (*Caretta caretta*) sea turtles e.g. [16,17]. Liebman and Granda found 3 photopigments in the green turtle retina absorbing maximally at 440 nm (SWS), 502 nm (MWS), and 562 nm (LWS) [18]. Recent evidence indicates that green turtles are also likely to have a fourth, ultraviolet sensitive (UVS) photo-pigment, like their freshwater relatives [17]. Turtles possess at least four different types of oil droplets, again indicating they have four spectral sensitivities, like birds [17]. Each type of oil droplet may be associated with a specific photopigment, or may combine with different photopigments to produce multiple cone receptor types [14,19].

Turtles, like many other vertebrates, also possess double cones, a specialized structure consisting of two cones joined together [20]. The function of the double cone is still unknown; however it has been hypothesized in both birds and reptiles to play a role in discriminating between levels of luminosity or brightness [20-23]. Although the exact composition of the double cone structure is unknown, in fresh water turtles both of the members that make up the double cone have LWS photoreceptors [19].

We created a chromatic space model of the green turtle visual system (sensu [24]) to investigate the following questions: Are green, hawksbill, and flatback turtles selectively ingesting particular types of debris over others, and if so, what characteristics of that debris (colour, texture, translucency, luminance, or background contrast) are most relevant to turtles’ foraging choices?

## Results

**Figure 1:**
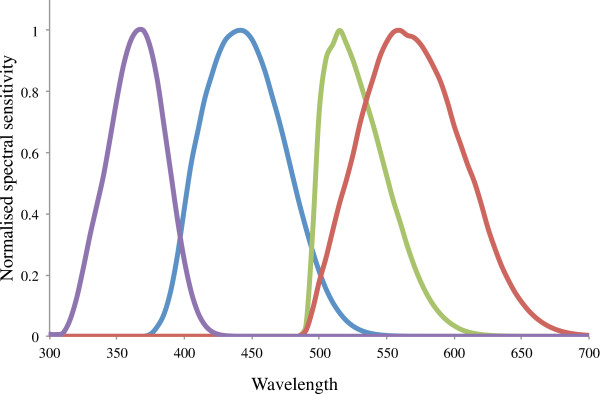
**Modelled spectral sensitivity of *C. mydas.*** Each peak represents the photopigment multiplied by the transmissivity of its associated oil droplet and by the ocular media.

Our visual model resulted in peak sensitivities of 365, 440, 515, and 560–565 (Figure 1). The mixed effects modelling results indicate that sea turtles select the debris they ingest based on a variety of physical properties. In fact, debris ingested by turtles was significantly different from beach debris for all environmental variables investigated with the exception of background contrast and the contribution of the UV cone (Table 1). Turtles differentiated items most strongly based on their luminance (p < 0.001, selectivity ratio = 0.640), flexibility (p < 0.001, selectivity ratio = 0.437), and translucency (p = 0.001, selectivity ratio = 0.290). Items ingested by turtles tend to be less bright (i.e. lower luminance value), more flexible, and more translucent than items found in the environment. With respect to wavelengths, items ingested by turtles had significantly lower short wavelength spectrum values (p < 0.001, selectivity ratio = 0.215).

**Table 1:** Model coefficients for physical factors influencing the selectivity of debris ingestion by sea turtles

	**Intercept**	**SE of intercept**	**Turtle effect**	**SD of turtle effect**	**p-value**	**Selectivity ratio**^**a**^
Flexibility	1.755	0.088	0.767	0.133	<0.001*	0.437
Translucency	1.295	0.069	0.375	0.104	0.001*	0.290
SWS	0.268	0.006	-0.058	0.010	<0.001*	0.215
MWS	0.291	0.005	0.028	0.008	0.002*	0.096
LWS	0.311	0.008	0.040	0.012	0.002*	0.127
UVS	0.130	0.007	-0.010	0.010	0.345	0.075
Contrast	25.981	1.551	-1.468	2.356	0.573	0.057
Luminance (sum of cones)	239.27	16.225	-153.144	24.569	<0.001*	0.640
Luminance (double cone)	74.978	4.930	-43.441	7.47	<0.001*	0.579

Note that the selectivity ratio indicates the relative strength of the turtles’ selectivity based on each factor. *indicate p values that are significant at the 0.05 level.

^a^Calculated as the absolute value of the ratio of the size of the turtle effect to the size of the intercept.

**Figure 2:**
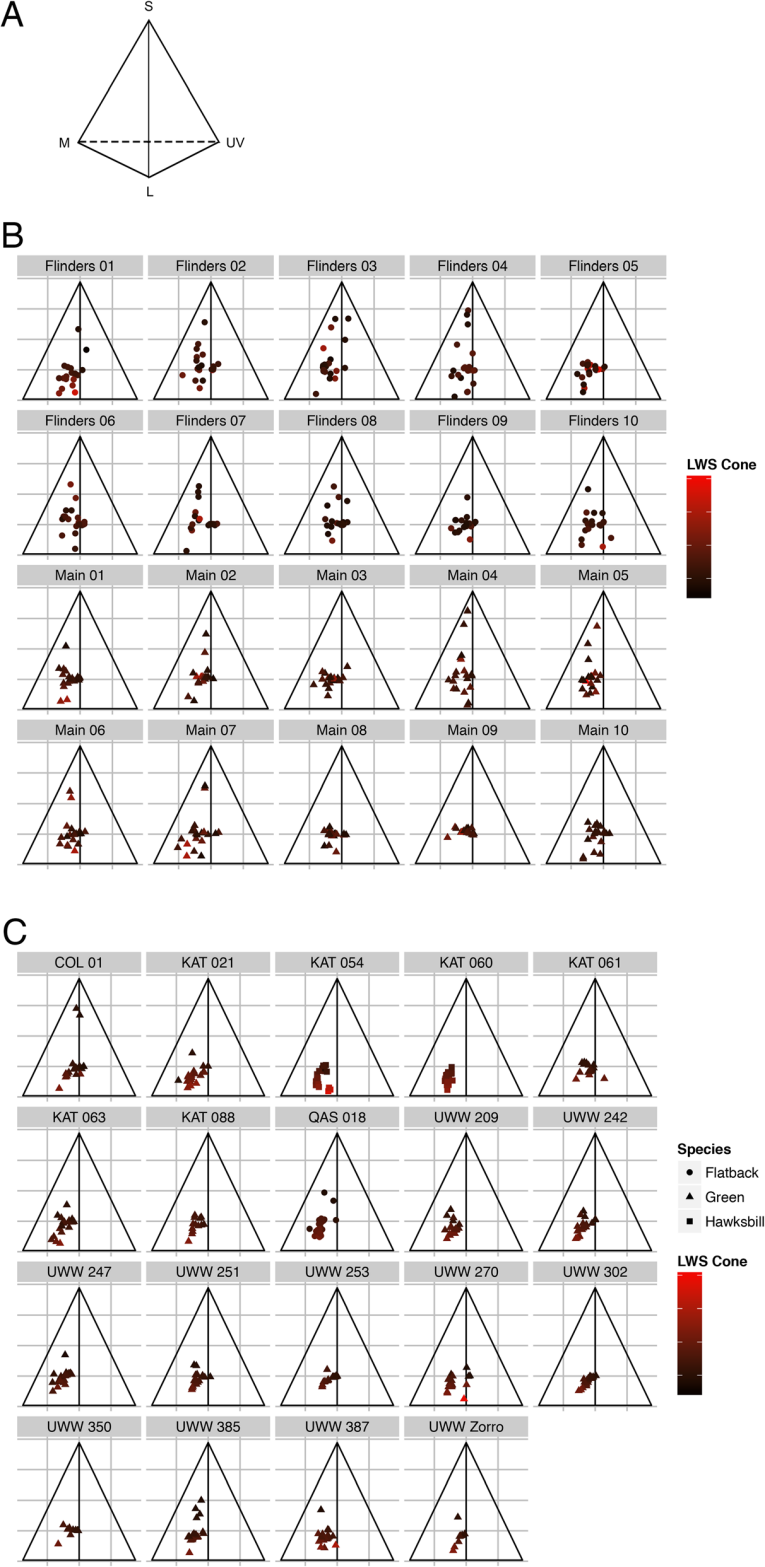
**Colour space triangles.** The visual space of a tetrachromatic sea turtle can be represented as a tetrahedron (2**A**). Each vertex represents the contribution from a different cone. The lower left corner is the medium wavelength cone, the lower right corner is the UV wavelength cone, and the top vertex is the short wavelength cone. In order to portray a 3 dimensional image in a 2 dimensional space, we use colour to represent the contribution from the fourth vertex, the LWS cone (red is a strong contribution from the long wavelength, black is not). We plot the plastic from each beach sample (2**B**) and turtle sample (2**C**) on a separate triangle. Every dot is a single piece of plastic, and the closer the dot to the vertex, the greater the contribution from that cone. n = 20 for all samples except KAT 88 (n = 13), UWW 242 (n = 19), and UWW 350 (n = 9).

A simple inspection of the turtle visual space models (Figure 2) shows the difference in the wavelengths of ingested debris and beach debris. The average value of debris ingested by turtles is lower in the short wavelength spectrum than that of beach debris, indicating that the items turtles eat are less blue than what is available to them in the environment.

There were no significant differences observed between plastics ingested by sea turtles of different life history stages (new recruits and juvenile turtles) with respect to the factors tested (colour, texture, translucency, luminance, and background contrast). However, hawksbill and flatback turtles did exhibit some significant differences compared to green turtles. Because we only had a small sample size for hawksbills (n = 2) and flatbacks (n = 1), we omitted them from analyses.

## Discussion

The spectral sensitivities we calculated (365, 440, 515 and 560–565) are well matched with previously published electroretinography (ERG) data of *C. mydas* spectral sensitivities. Levenson and colleagues [25] observed well-defined peaks at 515 and 570, with a relatively constant sensitivity below 500 nm; an earlier study found peaks at 450, 520, and 600 [18]. The technique of high frequency flicker ERG used by Levenson et al. [25] is likely more accurate in the longer wavelengths, as it more successfully isolates the cone response from the rod response. However, the turtles in this study were older than those used by Liebman and Granda and may have experienced a decline in short wavelength vision similar to elderly humans, explaining the lack of a defined short wavelength peak [25]. Our model, therefore, matches observed sensitivities based on ERG.

We assumed that beach debris was a reasonable proxy for ocean-borne debris in the nearshore area inhabited by these turtles, and therefore represents the debris “available” to the turtles. Although there are limitations of using beach debris as a proxy for ocean debris, it has been widely used in previous studies [26]. Thiel et al. [27] conducted a multi-year comparison of anthropogenic marine debris on beaches and in nearshore waters, finding the proportions of different items to be similar. Locally, an analysis of beach debris and nearshore trawl debris for locations around North Stradbroke Island found similar proportions of different colors of debris in both beach and trawl surveys (unpublished observations, Q. Schuyler). We are therefore confident that local beach debris is representative of nearshore ocean debris available to turtles analysed here.

It is clear from the statistical results, as well as from inspection of the turtle visual space data that turtles are selective in what they eat (Table 1, Figure 2). Turtles do not tend to ingest debris that is reflective in the short wavelengths; i.e. blue items. When turtle preferences were analysed based on a human categorical description of colour rather than a turtle visual space model, blue was similarly found to be less prevalent in turtle samples than in beach surveys [28]. Also in support of our findings, a laboratory-based study of loggerhead and Kemp’s ridley turtles indicated that both species avoided blue dyed bait [29].

Colour is not the only visual factor employed in food selection. In other animal species, contrast has been found to be as important or even more influential than colour in selecting food sources [30,31]. The fact that turtles are selecting against blue items could indicate that blue plastics are less readily visible against the blue background of the open ocean. We measured this contrast value by calculating the tetrachromatic distance between each debris item and a background measurement of open ocean water, but found that turtles did not selectively ingest items based on contrast. However, this may be partially due to limitations of the model. Similar models calculating colour space distances have reliably predicted honeybee behaviour when visiting orchid mimics. Bees were more likely to visit an orchid mimic when there was a small colour distance between the orchid and its preferred food source than when the colour space distance was large; in other words, when the mimic was a similar colour to their preferred food choice [32]. However, the honeybee model was only successful when incorporating second order visual processing, assuming interactions between photoreceptor types [33]. Our model did not incorporate these interactions, which may explain why turtles did not appear to select for high contrast items.

Turtles selected debris with significantly lower luminance values than those of beach debris, possibly because dark objects stand out better against the bright ocean background [34]. However, we cannot completely exclude the possibility that the prevalence of darker objects in the turtles is partially an artefact of our study design, as the debris in the turtles’ gastrointestinal system is exposed to digestive fluids and other waste, which may result in a reduction of luminance. Further work on clarifying the differences in selectivity between contrast and colour would help to elucidate these results.

The visual space model investigates colour and luminance, but other characteristics influence ingestion selectivity in turtles even more than colour. Turtles select plastics most strongly based on their flexibility and translucency. Our model suggests that turtles prefer highly flexible and translucent objects, both of which are key characteristics of one of their preferred natural prey items: jellyfish. This work demonstrates that turtles are indeed selective, and it also provides support for the widely postulated “jellyfish hypothesis”. Proper waste disposal, particularly for common end user items such as plastic bags and other soft, translucent items which are preferentially ingested by marine turtles, may help to reduce the rapidly increasing debris ingestion rates in threatened sea turtles. We hope this research can inform conservation efforts not only for endangered sea turtles, but we also suggest applying similar analyses for other visual predators to investigate the key factors that drive ingestion rates and anthropogenic debris selectivity.

## Conclusions

Using models to visualize how turtles “see” the plastic they ingest, we find strong support for the hypothesis that they ingest plastic because of its resemblance to a typical prey item, jellyfish. Our model can be extended to other species to better understand why wildlife consume plastic and to effectively focus conservation and remediation efforts.

## Methods

### Visual system model

We modelled the spectral sensitivity of the green sea turtle by incorporating measurements of the photopigments, oil droplets, and ocular media. We generated generic spectral photopigment curves [35-37] based on the peak absorbances for the three known green turtle photopigments: 440 nm, 502 nm, and 562 nm [18]. Since measurements of the green turtle UVS pigment have not been conducted, we simulated a UVS curve based on the UVS pigment of the freshwater turtle *Pseudomys scripta*. As freshwater turtles tend to have pigment maxima at longer wavelengths than sea turtles, we shifted the peak absorbance for the *Pseudomys* UVS curve 7 nm shorter to 365 nm [19].

For oil droplet measurements, we assumed that the orange oil droplets were associated only with photoreceptors containing the LWS visual pigments, yellow with the MWS, clear (UV-reflective) with the SWS pigments, and colourless (UV-transmissive) with the UVS photoreceptors. We used published curves for yellow and orange oil droplets from green turtles [18], and clear oil droplets from *Pseudomys* scripta [19]. We shifted the clear oil droplet spectrum shorter by 15 nm, corresponding to the difference in peak wavelength between the SWS pigments of *P. scripta* and of *C. mydas*[19]. We were unable to find published spectra for the UV-transmissive oil droplet in turtles, but as it has no significant absorbance above 325 nm, it would not affect the shape of the UV photopigment curve.

We applied the Hart correction to each oil droplet [38], converted to transmissivity, and multiplied the photopigment curve by the transmissivity of its associated oil droplet. We then multiplied the four resulting curves by the transmissivity of the ocular media [17] and normalized the result for each cone to an absorbance maximum of 1 to create a modelled spectral sensitivity curve for green sea turtles.

### Debris collection and measurement

We conducted necropsies on sea turtles stranded in southeast Queensland, Australia, between 2006 and 2013, and collected all pieces of debris that had been ingested by the animals (Table 2). For more details see [28]. Of 115 necropsied animals, nineteen had ingested sufficient quantities of debris for our analysis (16 green turtles, 2 hawksbill turtles, and 1 flatback turtle). To estimate the debris to which animals would have been exposed we conducted ten beach surveys on each of two different ocean-facing beaches on North Stradbroke Island (Flinders Beach and Main Beach) between 2011–2013 (for detailed methodology see [28]). All items of anthropogenic debris over 5 mm in length between the water line and the dominant vegetation line were collected in a 100 m transect. We selected 20 random debris subsamples from each beach and each turtle sample. Three of the turtles had ingested fewer than 20 items of debris, so for these turtles, all pieces were analysed.

**Table 2:** Characteristics of necropsied turtles

	**All turtles necropsied**	**Turtles with debris**
Species		
Green	88	16
Hawksbill	24	2
Flatback	1	1
Loggerhead	2	0
Size class		
Pelagic (CCL < 35 cm)	22	12
Benthic (CCL > 35)	93	27

We assigned each piece of debris a measurement of flexibility between 1 (impossible to bend without breaking) and 3 (easily malleable). We also assigned a measure of translucency between 1 (completely opaque) and 3 (possible to read text through the item). We chose translucency and flexibility because they are visual characteristics in addition to colour which might be used for prey selection. Using an Ocean Optics JAZ spectrophotometer we measured the reflectance of each item between 300–800 nm wavelength. In 49 of the plastic samples we did not dark-calibrate the spectra, so some of the reflectances were slightly below zero. To each of the measurements for these samples we added a constant value (equal to the largest negative value for the sample) in order to ensure that the minimum value was non-negative. Because the negative values were quite small with respect to the maximum reflectances, and represent only a linear shift, this correction factor did not affect the outcome of our modelling.

We used our calculated green turtle spectral sensitivities to model how each item of debris would appear in the turtles’ visual space [39]. Because there are virtually no studies on the visual systems of hawksbill and flatback turtles (but see [40]), we used the green turtle spectral sensitivity curves (as modelled above) for all species. The visual space for a tetrachromatic animal can be represented as a three dimensional tetrahedron with one vertex for each cone. Plotting the relative excitation of each photoreceptor within this space generates a representation of the colour of an object as it would appear to a turtle’s visual system.

Using the Vorobyev-Osorio noise-limited chromatic space model [41] we also calculated the three-dimensional distances between each piece of debris and a measurement of background colour that turtles would be likely to encounter; open ocean water. This gives an indication of the contrast of each item to the background colour. This calculation relies on an estimate of the proportions of cones present in the retina. Although these data are not known for sea turtles, the proportions of oil droplets are [17], so we assumed the proportions of cones in the retina to be equal to the proportions of oil droplets associated with them. Finally, we calculated two different measures of luminance. For the first we added the total reflectance values for all four cones. Since the double cone may be responsible for luminance discrimination, we calculated a second measurement using the total reflectance of the LWS cone only [19].

In order to determine whether turtles exhibited a selectivity for debris based on the physical characteristics measured (colour, texture, translucency, luminance, and background contrast), we used linear mixed effects models (R version 3.0.1, package lme4) [42] with the physical factors as response variables, and the location the plastic was found (turtle or beach) as the predictor variable. In order to control for autocorrelation among plastic items within a beach or stomach sample, we incorporated a random effect for each beach or turtle sample. We also investigated the differences between species and life history stages of turtles with respect to each physical characteristic. Because of the complex nature of the data set, we analysed each factor separately. In order to obtain a relative measurement of the strength of each term, we calculated the absolute value of the ratio of the effect size to the intercept term. Note that the larger the ratio, the more highly selective the turtles are for the variable.

### Ethical statement

Because this research was carried out on dead stranded sea turtles, no ethical approval was required.

## Competing interests

The authors declare that they have no competing interests.

## Authors’ contributions

QS carried out the field and lab work and drafted the manuscript. CW assisted in statistical analysis. KT and JM conceived of the study. BDH contributed substantial editing of the manuscript. JM participated in the design and coordination of the study. All authors read and approved the final manuscript.
